# Functional transformations of odor inputs in the mouse olfactory bulb

**DOI:** 10.3389/fncir.2014.00129

**Published:** 2014-11-04

**Authors:** Yoav Adam, Yoav Livneh, Kazunari Miyamichi, Maya Groysman, Liqun Luo, Adi Mizrahi

**Affiliations:** ^1^Department of Neurobiology, Institute of Life Sciences, The Edmond and Lily Safra Center for Brain Sciences, The Hebrew University of JerusalemJerusalem, Israel; ^2^Department of Biology, Howard Hughes Medical Institute, Stanford UniversityStanford, CA, USA

**Keywords:** olfactory bulb, functional organization, neuronal populations, *in vivo* imaging, calcium imaging

## Abstract

Sensory inputs from the nasal epithelium to the olfactory bulb (OB) are organized as a discrete map in the glomerular layer (GL). This map is then modulated by distinct types of local neurons and transmitted to higher brain areas via mitral and tufted cells. Little is known about the functional organization of the circuits downstream of glomeruli. We used *in vivo* two-photon calcium imaging for large scale functional mapping of distinct neuronal populations in the mouse OB, at single cell resolution. Specifically, we imaged odor responses of mitral cells (MCs), tufted cells (TCs) and glomerular interneurons (GL-INs). Mitral cells population activity was heterogeneous and only mildly correlated with the olfactory receptor neuron (ORN) inputs, supporting the view that discrete input maps undergo significant transformations at the output level of the OB. In contrast, population activity profiles of TCs were dense, and highly correlated with the odor inputs in both space and time. Glomerular interneurons were also highly correlated with the ORN inputs, but showed higher activation thresholds suggesting that these neurons are driven by strongly activated glomeruli. Temporally, upon persistent odor exposure, TCs quickly adapted. In contrast, both MCs and GL-INs showed diverse temporal response patterns, suggesting that GL-INs could contribute to the transformations MCs undergo at slow time scales. Our data suggest that sensory odor maps are transformed by TCs and MCs in different ways forming two distinct and parallel information streams.

## Introduction

Most neural circuits in the brain are spatially organized, but the organization of the olfactory system has no clear continuous topography (Luo and Flanagan, 2007). Olfactory receptor neurons (ORNs) expressing the same receptor appear to be distributed randomly at the epithelium, but their axons coalesce into discrete units called glomeruli in the main olfactory bulb (OB; Mombaerts, 2006). The precise distribution of glomeruli on the surface of the OB forms a map of ORN inputs. Since ORNs respond to multiple odors, each odor is represented by a spatially distinct combination of active glomeruli (Mori et al., 2006; Soucy et al., 2009; Ma et al., 2012). The functional organization of this glomerular map has been shown to be grossly chemotopic (Mori et al., 2006). However, at a fine scale, neighboring glomeruli often have highly diverse odor response profiles (Soucy et al., 2009; Ma et al., 2012). Despite the wealth of knowledge on the formation and physiology of glomerular maps (Mori et al., 2006; Soucy et al., 2009; Murthy, 2011; Ma et al., 2012), any spatiotemporal transformations that these maps may undergo are poorly understood.

Olfactory receptor neurons inputs are conveyed downstream of the OB mainly by two populations of projection neurons—mitral cells (MCs) and tufted cells (TCs). Anatomically, both MCs and TCs send a single dendritic tuft into a single glomerulus. Mitral cells project their axons dispersedly to numerous cortical regions (Miyamichi et al., 2011; Igarashi et al., 2012), while TCs project densely to more focal targets, primarily in anterior regions of the cortex (Nagayama et al., 2010; Igarashi et al., 2012). While MCs form a monolayer in the deeper parts of the OB, TCs are scattered all along the external plexifrom layer (EPL) and include also a subpopulation in the deeper parts of the glomerular layer (GL) called external tufted cells (eTCs). External tufted cells have been extensively studied *in vitro* showing that they convey information locally in the OB (Hayar et al., 2004; Wachowiak and Shipley, 2006; Gire et al., 2012) and presumably also distally to the cortex (Igarashi et al., 2012). In addition to eTCs, the GL contains diverse types of interneurons (GL-INs). These include different types of periglomerular neurons (PGNs), which perform intra-glomerular feed-forward and feedback inhibition onto ORNs and MCs (Wachowiak and Shipley, 2006; Gire and Schoppa, 2009; Shao et al., 2009). Glomerular interneurons include also short-axon cells (SA) forming interglomerular lateral connections. Glomerular interneurons and eTCs are thought to support transformations such as gain control and pattern decorrelation (Cleland, 2010; Murthy, 2011; Friedrich, 2013). Recently, both electrophysiology and imaging were used to study odor responses of GL-INs (Tan et al., 2010; Homma et al., 2013; Kikuta et al., 2013) but those focused only on single glomerular modules and couldn’t differentiate interneurons and eTCs.

Odor map transformations have been described in flies and fish (Yaksi et al., 2007; Friedrich, 2013; Wilson, 2013). In mammals, however, spatiotemporal transformations of glomerular maps remain largely unexplored. We combined viral labeling with *in vivo* two-photon calcium imaging to functionally map three neuronal populations, TCs, GL-INs and MCs. We mapped odor responses of ~2000 single cells from these three neuronal populations and revealed their functional organization at a spatial scale ranging from microns to millimeters and their unique temporal dynamics at short and long time scales. Our data reveal unique characteristics of each population in both spatial and temporal dimensions.

## Methods

### Animals

We used C57BL/6 or Thy1-GCaMP3 (Chen et al., 2012) male mice (10–14 weeks old at the surgery). Animal care and experiments were approved by the Hebrew University Animal Care and Use Committee.

### Cloning, lentivirus production and AAV vectors

To generate lentiviral vectors we sub-cloned GCaMP3.0 (kind gift of L.L. Looger) into lentivirus (LV) transfer plasmid. We produced high titer Lentivirus particles as described elsewhere (Adam and Mizrahi, 2011). Briefly, we transfected human embryonic kidney cells (HEK293) with third-generation LV plasmids using polyethylenimine. The medium was collected after 36 h and after additional 24 h. Virus was concentrated using ultracentrifugation and resuspended in PBS. AAV1-hSyn-GCaMP3.0 was purchased from the University of Pennsylvania Gene Therapy Program Vector Core. AAV5-CKII-GCaMP5.0 was custom made at the University of North Carolina Gene Therapy Program Vector Core.

### Virus injection and chronic window implantation surgery

Mice were restrained using a low dose i.p. injection of Ketamine (10 mg/kg) and Medetomidine (0.083 mg/kg). Anesthesia was maintained with isofluorene (1–1.5%) and monitored using the pinch withdrawal reflex. The skull covering both OBs was exposed, cleaned and thoroughly dried. 3 mm round craniotomy was opened over both OBs using a 3 mm biopsy punch (Miltex, York, PA), and the bone was carefully removed.

Virus was stereotaxically injected directly into the dorsal OB using pressure at 2–3 locations in each OB. AAV1 was injected at 30° angle and depth of 0–200 µm from the surface, and released in small doses (~25 nl) every 25 µm. Lentivirus and AAV5 were injected perpendicular to the surface and released in small doses (~25 nl) every 30 µm at depth of 0–350 µm. The surface of the brain was thoroughly rinsed, and covered directly with a 3 mm diameter round coverglass (Menzel-Glaser, Braunschweig, Germany). The margin between the coverglass and the bone was gently sealed with histoacryl glue (B.Braun, Tuttlingen, Germany). Once dried, dental cement was applied around the window, to strengthen the seal and support the water during imaging. For repeated imaging, 0.1 g metal bar was glued to the skull (Livneh et al., 2009; Adam and Mizrahi, 2011). After surgery, mice received dextrose-saline subcutaneously and treated with carprofen (0.004 mg/g, s.c). All animals were allowed to recover at least 4 weeks post surgery.

In a few experiments, MCs data was collected through an acute imaging window. In those animals, AAV5 was injected through a small incision in the bone, sealed with agarose and dental cement. At the day of imaging we constructed an acute imaging window as described before (Livneh et al., 2009).

### Two-photon calcium imaging

Imaging was performed under ketamine/medetomidine anesthesia (100 mg/kg and 0.83 mg/kg, i.p.). Imaging lasted up to 10 h per session. We assessed the depth of anesthesia by monitoring the pinch withdrawal reflex and added ketamine/medetomidine when needed. We continuously monitored the animal’s rectal temperature and maintained it at 37 ± 1°C. Each OB was imaged several times to collect as many neurons as possible for mapping, usually two consecutive imaging sessions. In some animals we mapped both OBs (i.e., four sessions per animal). To collect the “external” odors dataset (Figure 5), some animals underwent an additional imaging session. Animals were placed under the microscope in a custom-made stereotaxic device via the metal bar and kept in fixed angle relative to the objective.

**Figure 1:**
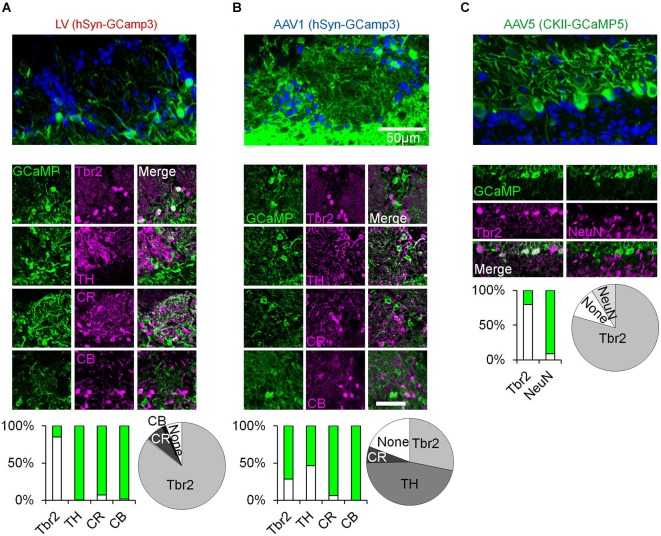
**Immunohistochemical analysis of OB cell types labeled with distinct viral vectors. (A–C)** Top, confocal micrographs from the OB following virus injection. Middle, representative images of GCaMP expression and its overlap with Tbr2 (Winpenny et al., 2011), TH, Calretinin (CR), and Calbindin (CB) positive neurons (Parrish-Aungst et al., 2007; Adam and Mizrahi, 2011). Green images—expression of GCaMP. Magenta images—Staining with antibodies against the indicated marker. Merged images—double labeling of GCaMP and the respective marker. Bar graphs—percent of double labeling between GCaMP and the indicated marker (Green = GCaMP positive and marker negative, white = GCaMP positive and marker positive). Pie charts—summary of the composition of each sample (based on the bar graphs). LV—Lentivirus, AAV—adeno-associated virus, hSyn—human synapsin promoter, CKII—CamKII promoter. Tbr2—T-Brain gene 2, TH—tyrosine hydroxylase, CR—calretinin, CB—calbindin, NeuN—neuronal nuclear marker.

**Figure 2:**
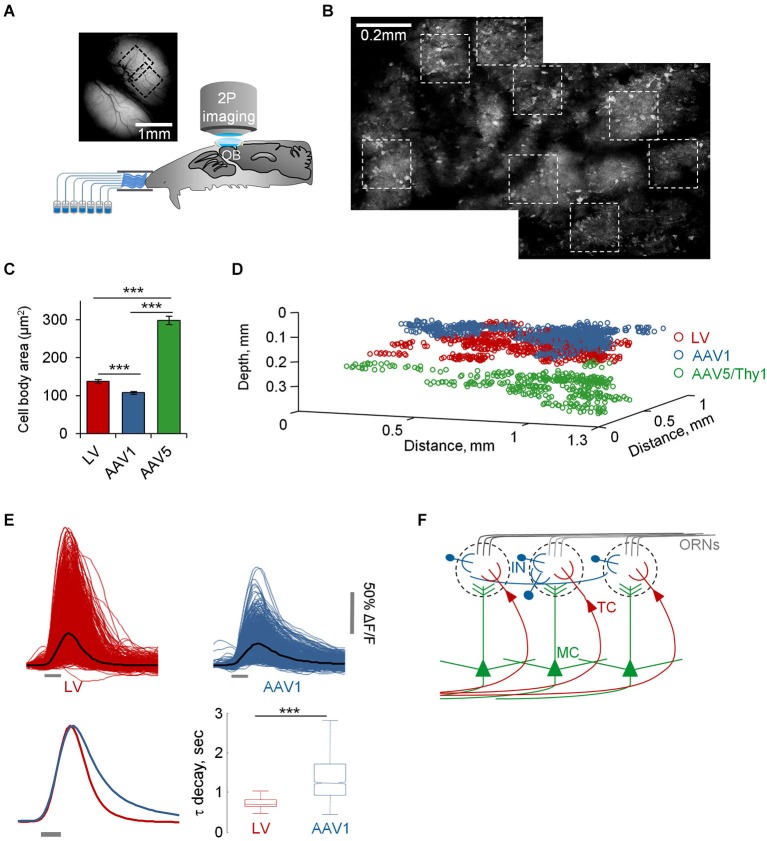
**Functional mapping of TCs, GL-INs and MCs. (A)** Experimental setup. Schematic of an anesthetized mouse implanted with a chronic imaging window placed under the two-photon microscope. Mice were stimulated with seven odorants, delivered through an olfactometer with completely separate channels. The image is a still image of both bulbs in a representative chronic window. **(B)** Two consecutive low resolution two-photon micrographs of cells expressing GCaMP3 after infection with AAV1-hSyn-GCaMP3. Images are from the same OB shown in **(A)** (where imaged regions on the blood vessel map are denoted by black dotted squares). In this OB, eight imaging fields were used for imaging (white dotted squares). **(C)** Quantification of the 2D area of cell bodies labeled and imaged with the different viruses (mean ± S.E.M, *n* = 91 TCs, *n* = 79 GL-INs, *n* = 55 MCs, ***p < 0.001, *T*-test). **(D)** Depth from the surface of all cells imaged in this study (TCs—130 ± 26 µm, GL-INs—82 ± 21 µm, MCs—270 ± 41 µm). Locations of cells are aligned within mice. **(E)** Top, Calcium transients of all cell-odor pairs from LV and AAV1 infected neurons (black line = mean). Bottom left, normalized mean transients. Bottom right, Distribution of the mean τ decay of all cells. Tufted cells decays were faster and homogenous. In agreement with the heterogeneous nature of the GL-INs, their τ decays were highly heterogeneous and significantly slower compared with TCs (*n* = 298 TCs and *n* = 189 GL-INs). Box shows the 25 to 75 percentile, line = median, bars show the data limits. *** *p* < 0.001, unpaired *T*-test. **(F)** Scheme of the OB circuit and the cell types studied in this work.

**Figure 3:**
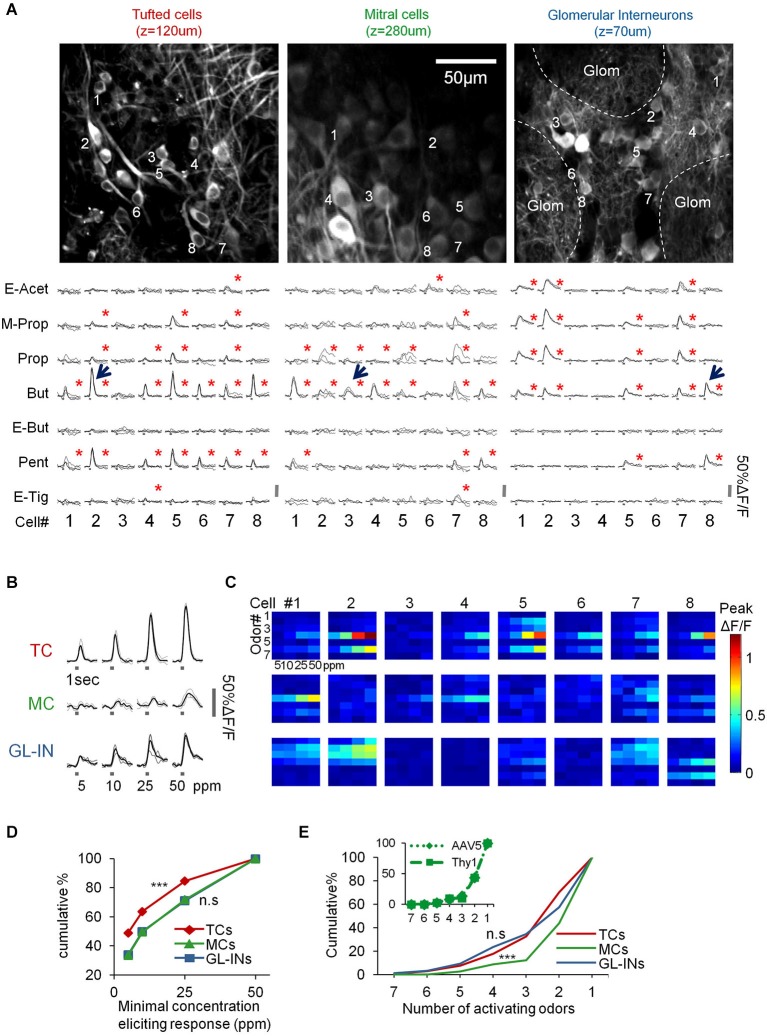
**Basic response properties of TCs, GL-INs and MCs. (A)** Two-photon micrographs of fields used for calcium imaging, and a sample of calcium transients elicited in eight representative neurons in the field (cells marked by numbers) by the different odorants (at 50 ppm). Gray traces—single trials, Black traces—mean response. Stimulus duration = 1 s. Red asterisk = statistically significant response (see Section Methods). **(B)** Examples of responses to four concentrations of butanal in single cell odor pairs from each population (cell-odor pairs are denoted by arrows in **(A)**). Gray traces—single trials, Black trace—average. **(C)** Full response profiles of the same cells shown in **(A)** (for a total of 24 neurons). Rows = odors, columns = concentrations. Colorcode = max ΔF/F. **(D)** Response thresholds, cumulative graphs of the lowest concentration eliciting response in each cell. Tufted cells are activated at significantly lower concentration (***p < 0.001, *T*-test). **(E)** Odor selectivity. Cumulative graphs of the number of activating odors (only for cells responding to at least one odor). Mitral cells response profiles are sparser compared to TCs and GL-INs (***p < 0.001 *T*-test). Inset, selectivity of MCs labeled with AAV5-GCaMP5 (dotted line) vs. Thy1-GCaMP3 (dashed line).

**Figure 4:**
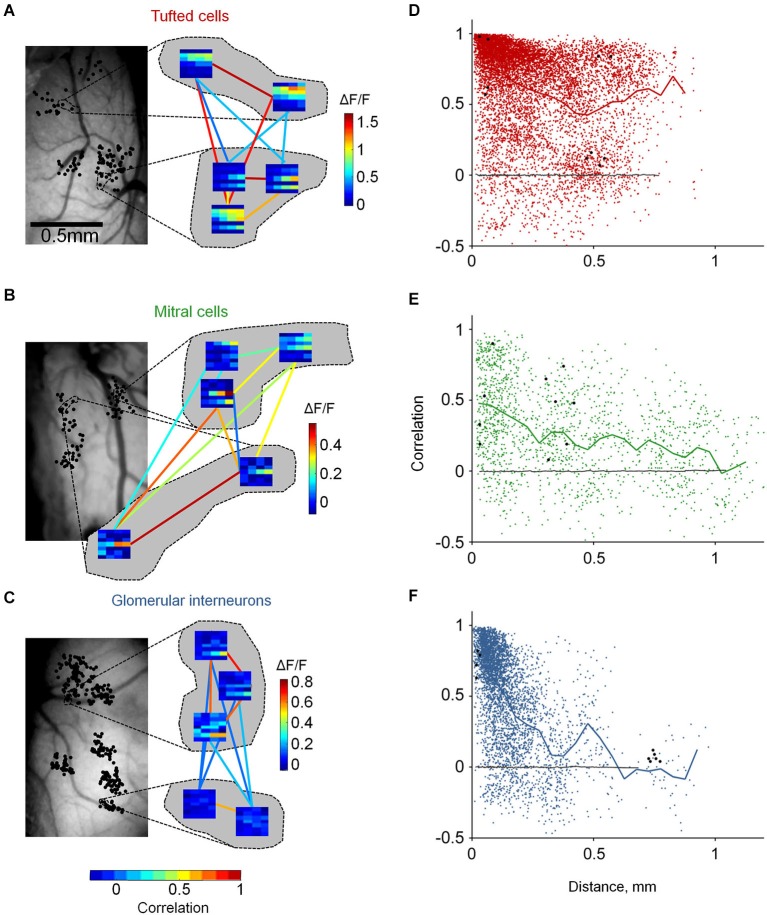
**Distinct functional architecture of TCs, GL-INs and MCs. (A)**—TCs, **(B)**—MCs, **(C)**—GL-INs. Left, still image of the blood vessels on the dorsal surface of the OB. Black dots denote the location of all imaged cells (in a 2D dorsal view). Dashed lines mark the location of the cells which their enlarged response profiles are shown on the right. Right, examples of response profiles from five neurons in each OB. Each row represent one odor (1-E-Acet, 2-M-Prop, 3-Prop, 4-But, 5-E-But, 6-Pent, 7-E-Tig), and each column represents a single concentration (5, 10, 25, 50 ppm). Color-code (vertical) is the max ΔF/F in each condition. Colored lines represent the signal correlation between the connected cells, represented by the horizontal color bar. **(D)**—TCs, **(E)**—MCs, **(F)**—GL-INs. Signal correlation between the response profiles of all pairs as a function of the distance between the cells. Thick line—mean correlation averaged in 50 µm bins. Black dots—the example pairs from **(A) (B)** or **(C)**. Black line—correlation between randomly shuffled response profiles. TCs: *n* = 8755 pairs from *n* = 306 active neurons in six OBs, GL-INs: *n* = 4128 pairs from *n* = 194 active neurons in six OBs, MCs: *n* = 1682 pairs from *n* = 149 active neurons in 10 OBs.

**Figure 5:**
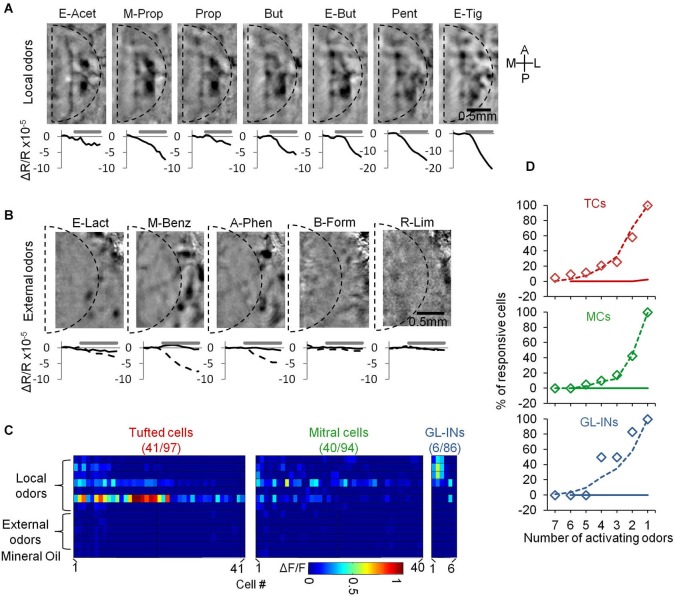
**Neurons from the dorsal surface are not activated by distant glomeruli. (A)** Intrinsic signal imaging maps showing activation by the panel of odorants used in this study. The borders of a standard chronic imaging window are denoted by the dashed line. Beneath each map we show the dynamics of the intrinsic signal before and during a 4 s stimulus. Signal is the sum intrinsic signal within the dashed line. **(B)** Same as in **(A)** but for five “external” odors. Curves in the bottom shows the dynamics of the intrinsic signal within the window (black) and outside the window (dashed). **(C)** Calcium responses of all responsive neurons that were tested with both “local” and “external” odor sets. Color-code—Peak ΔF/F. Cells in each population are sorted according to the number of activating odorants. Only the responsive cells in each group are presented (total numbers are outlined in the title of each panel). **(D)** Cumulative graphs of the number of activating odors in each population (responsive cells only). Diamonds—local odors from this dataset, dashed line—local odors in the main dataset (same as Figure 3E), solid line—external odors.

We imaged the OB using an Ultima two-photon microscope from Prairie Technologies (Middleton, WI, USA), equipped with a 16X water-immersion objective (0.8 NA, Nikon). We delivered two-photon excitation (950 nm) with a DeepSee femtosecond laser (Spectraphysics), and collected low magnification Z-stacks (fields of 676 × 676 µm) at 1–2 µm resolution at the Z dimension. These were used to locate high magnification imaging fields and calculate the 3D distances between the cells. We then zoomed-in at different regions (up to 8 fields per OB), based solely on the optical clarity. Size of imaging fields was 169 × 169 µm, and we collected images of 420 × 210 pixles at acquisition rate of ~7 Hz. Since MCs had much larger soma size (Figure 2A), we collected part of the MCs data from larger fields (up to 250 × 250 µm) at the same pixel size. We imaged in cycles of 120 frames for every 1 s odor stimulus and 200 frames for 15 s odor stimulus. Imaging cycles were triggered by the odor delivery system, as described below.

### Odor delivery

**Figure 6:**
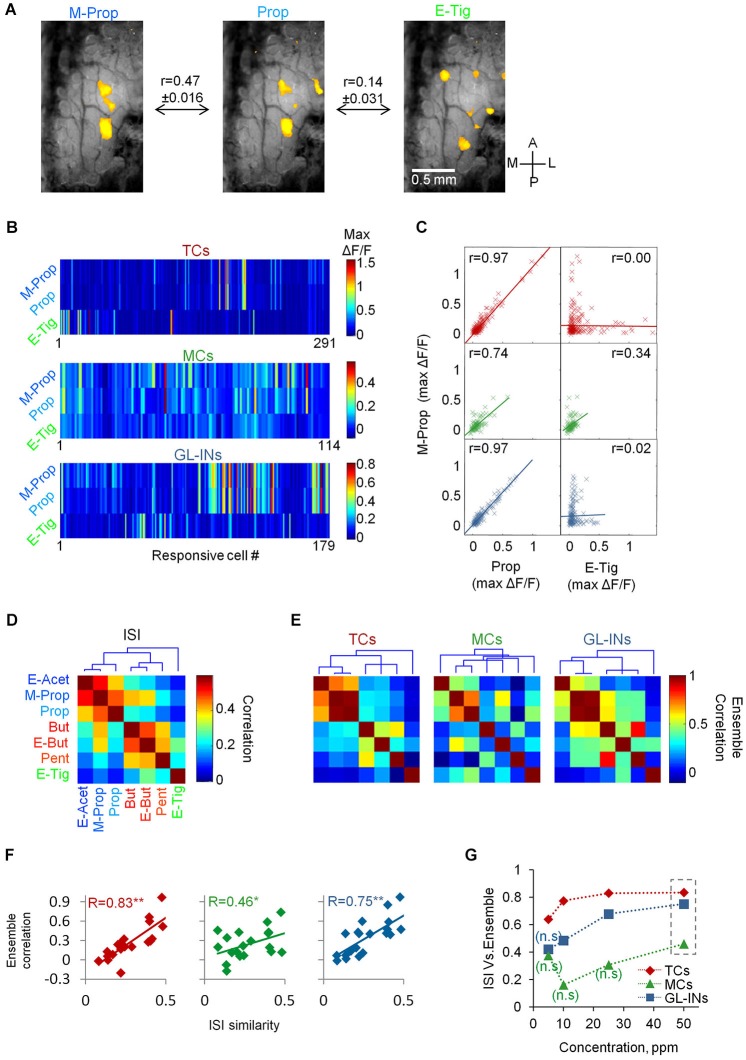
**Input-output relationships of TCs, GL-INs and MC ensembles. (A)** Representative intrinsic signal imaging maps in response to three different odors from one OB. Maps were filtered to represent ORNs input (see Section Methods). *r* = Pearson correlation between the intrinsic signal maps across the data (mean ± S.E.M, *n* = 36 OBs; *n* = 252 maps). **(B)** Ensemble calcium activity for three odors in the three neuronal populations. Only responsive cells are shown (TCs, *n* = 291 cells, GL-INs, *n* = 179 cells, MCs, *n* = 114 cells). **(C)** Max ΔF/F for M-Prop as a function of max ΔF/F for Prop (left column) or E-tig (right column). *r* = correlation between the ensemble responses to the odor pairs. **(D)** Correlation of all odor pairs at the ORN level, calculated as in **(A)** from the intrinsic signal maps. Dendrogram shows hierarchal clustering of the correlation values. The three clusters are represented by color in the odor names (blue, red, and green). ISI—Intrinsic signal imaging. **(E)** Ensemble correlation for all odor pairs in the three neuronal populations. Dendrograms show hierarchical clustering of the correlation values. Odors as in **(D)**. The intrinsic signal clusters are preserved in the TCs but not in the MCs. **(F)** Ensemble correlation of each population at 50 ppm concentration (as in **C** and **E**) were plotted as a function of the correlations between the ORNs input (as in **A** and **D**). *r* = Pearson correlations between both vectors. **p < 0.01, *p < 0.05. ISI—Intrinsic signal imaging. **(G)** Correlation between the intrinsic signal similarity and the ensemble correlation (as presented in panel **F**) as a function of the odor concentration (n.s–non-significant correlation values).

To deliver odorants we used a custom-made 7-channel olfactometer. To avoid cross-contamination between odorants we used separate tubing for each channel (see Figure 2C). For odor delivery, we switched a N_2_ flow into one of the odor vials for the desired duration, while keeping the overall flow constant. We used a panel of seven odorants that activate different and partially overlapping areas in the dorsal OB (ethyl-acetate, butanal, pentanal, ethyl-tiglate, propanal, methyl-propionate and ethyl-butyrate; from Sigma-Aldrich; Figures 5, 6). We diluted the odorants in mineral oil according to their individual vapor pressures to give a nominal headspace concentration of 1000 ppm. We further diluted the odorants by N_2_ flow of 10–100 ml/min, mixed with N_2_ flow of 900–990 ml/min, and O_2_ flow of 1000 ml/min. This procedure achieves final concentration of 5–50 ppm. The flow of the two N_2_ channels was controlled using mass flow controllers (M100B, MKS Instruments, Andover, MA, USA). Odorants were presented for 1 s at 5, 10, 25 and 50 ppm (22 s inter-stimulus interval). Each protocol included 28 stimuli in pseudo-random order of odors and concentrations, and was repeated four times in each imaging field. Additionally, in each field we delivered a protocol of the seven odorants for 15 s at 50 ppm (36 s inter-stimulus interval, four repetitions). Normally we used for analysis all four repetitions, in rare cases we had to discard one trial. Two-photon excitation and image collection were triggered 3–5 s before the stimulus onset and lasted ~17 s or ~28 s (1 or 15 s respectively).

We monitored the animal’s respiration using a low pressure sensor (1-INCH-D1-4V-MINI, “All sensors”). We connected the sensor to a thin stainless steel tubing (OD 0.7 mm) which we placed at the animals’ contra-lateral nostril. The information from the pressure sensor was passed to an analog converter (window discriminator), which was used to identify the inhalation onset during the respiratory cycle, and trigger odor delivery at the inhalation onset.

### Intrinsic signal imaging

Intrinsic signal imaging of the dorsal surface of the OB was performed using an Imager 3001 (Optical Imaging) *via* thinned bone, as described by Livneh et al. (2009). Briefly, mice were anesthetized using ketamine/medetomidine (100 mg/kg and 0.83 mg/kg, i.p.), and carprofen (4 mg/kg). Depth of anesthesia was assessed using the pinch withdrawal reflex. We monitored the animal’s rectal temperature and maintained it at 36 ± 1°C. The surface blood vessel pattern was acquired under green light illumination (546 nm). Light reflectance from the surface of the OB (630 nm light illumination) was captured using a CCD camera (Dalsa 1M60P). Images were acquired with a spatial resolution of ~10 µm/pixel. Images of 1024 × 1024 pixels were binned (3 × 3) for analysis.

We analyzed intrinsic signal maps offline, using custom written software in Matlab. We obtained the normalized intrinsic signal by Δ*R/R* = (*R*_odor_ − *R*_air_/*R*_air_; where *R*_odor_ is the intrinsic signal during the last 2 s of a 4 s odor presentation, and *R*_air_ is the intrinsic signal during the 2 s before odor presentation. Intrinsic signal maps were the averaged response to the odorant in 4–8 trials. We filtered the Δ*R/R* image to remove contamination from a large-scale hemodynamic signal by subtracting a copy convolved with a Gaussian spatial kernel (STD = 315 µm). For quantitative analysis, we set the threshold for activation at 1.65 s.d. above the mean signal. This image processing yields intrinsic signal maps that reflect mostly ORN input to the OB (e.g., Uchida and Mainen, 2003; Soucy et al., 2009).

### Immunohistochemistry and confocal microscopy

We perfused mice transcardially with PBS followed by 4% paraformaldehyde, and soaked the brains in 30% sucrose. We sectioned OBs coronally (30 µm) on a sliding microtome and performed immunohistochemsitry on floating sections in 5% heat-inactivated goat serum and 0.5% triton in PBS. We used the following primary antibodies: rabbit anti-calretinin (CR) (1:2000) and mouse anti-calbindin (CB) (1:1000) (Swant, Bellinzona, Switzerland), mouse anti-Tyrosine Hydroxylase (TH) (1:500) (Immunostar, Hudson, WI, USA), mouse anti-Tbr2 (1:100) (Abcam, Bristol, UK) and mouse anti-NeuN (Millipore, Billerica, MA, USA). GCaMP was amplified using chicken anti-GFP (1:1000, Millipore). The following secondary antibodies were used: biotinylated goat anti-rabbit (1:500), DyLight549 conjugated goat anti-mouse (1:500) and DyLight488 conjugated goat anti-chicken, (Jackson ImmunoResearch, West Grove, PA, USA). Amplification was carried out using Cy5 conjugated streptavidin (Jackson ImmunoResearch). Slices were imaged with a SP50 confocal microscope, via a 60X (1.4 NA) oil objective (Leica, Wetzlar, Germany). Counting of neuronal somata was carried out manually using ImageJ.

### Data analysis

We performed data analysis using ImageJ^1^ followed by custom routines written in Matlab (The Mathworks). Regions of interest (ROIs) corresponding to individual cell bodies were manually drawn; the mean fluorescence of each cell body was extracted by imageJ and used for analysis. Odor delivery was triggered at the inhalation onset (see above), and all trials were aligned according to the frame imaged at odor onset (defined as time = 0). Relative fluorescence change (Δ*F/F*) was calculated, baseline fluorescence (*F*_0_) was the mean fluorescence over 1 s before odor onset. Δ*F/F* traces were low-pass filtered using a finite impulse response filter (1 s time window, cutoff = 0.2). Zero-phase filtering was achieved by two passes of the low-pass filter using the Matlab filtfilt function.

To identify responsive cells, we subtracted the filtered Δ*F/F* trace from the original Δ*F/F* trace, resulting in the “baseline noise” trace. We then defined a response window equal to the stimulus duration +2 s (3 or 17 s window), and converted the filtered traces to Z-scores by dividing them with the s.d. of the baseline noise along this window. We applied this procedure also to the mean trace of each condition. To define threshold for response, we used a dataset of cells from all three populations stimulated with pure mineral oil (as part of the “external odors” dataset, Figure 5). We pooled all the sampling points from these cells during the response window of the mineral oil trials from each population. These values distributed normally (not shown), and we defined the threshold for each population as the mean +1.96 s.d. of this distribution. Using this procedure we defined threshold Z-score value for each population and stimulus duration (1 s/15 s): TCs, *Z* = 2.73/2.81, GL-INs, *Z* = 2.58/2.78, MCs, *Z* = 3.00/3.56. Similar analysis yielded different thresholds for the mean traces: TCs, *Z* = 2.78/3.24, GL-INs, *Z* = 1.55/1.9, MCs, *Z* = 1.84/2.99. We considered a trial as responsive if the Z-score along the window was >threshold for at least three consecutive frames. A cell was considered responsive if it was responsive for at least two trials and for the mean trace.

Response magnitude was defined as the peak Δ*F/F* along the response window, averaged between all trials. For signal correlation (S.C.) analysis we used the response profile of a cell i.e., the peak Δ*F/F* for seven odors at four concentration (1 s stimuli). Signal correlation is the Pearson correlation between the response profiles (vectors of 28 values) of a pair of cells. We calculated S.C. between all cell pairs from single OBs. We included in this analysis only cells that responded to at least one stimulus. To confirm the robustness of this analysis we repeated it after eliminating cells with strong responses, which did not qualitatively impact the results (data not shown).

To calculate ensemble correlations, each population was organized as 28 vectors with the responses of all cells to a condition (odor × concentration). We then calculated the Pearson correlation between all possible pairs of ensembles (21 combinations in each concentration). In each concentration we included only cells that were responsive to at least one odor. Similarly we calculated the similarity between the intrinsic signal maps. Pixels in filtered intrinsic signal maps (see above) were rearranged as vectors. We calculated the Pearson correlation between the maps of all the seven odors (21 combinations) per OB. We repeated this calculation for *n* = 36 OBs, and then calculated the average value for each pair of odors. Hierarchal clustering of the correlation values was performed using the Matlab function linkage. To confirm the robustness of the comparison between the intrinsic signal similarity and the ensemble correlations, we repeated it 10 times after randomly eliminating either 50% of the cells or 50% of the intrinsic signal imaging maps and also repeated it after eliminating cells with strong responses. All eliminations did not qualitatively impact the results (data not shown). This analysis also validated that although at lower odor concentrations we used smaller number of cells for analysis (since we used only responsive cells), the dataset was big enough and it did not affect the conclusions.

## Results

### GCaMP expression in distinct OB cell types

To characterize odor responses of different neuronal populations of the OB we used a viral approach to express genetically encoded calcium indicators. We chose GCaMP3 (Tian et al., 2009) and GCaMP5 (Akerboom et al., 2012) as these were previously shown to be reliable reporters of spiking activity in various brain regions including the OB (Tian et al., 2009; Akerboom et al., 2012; Kato et al., 2012; Wachowiak et al., 2013). After testing different viruses and promoters (not shown), we chose three variants that expressed the indicator in three largely distinct neuronal populations due to a combination of promoter expression and viral tropism (Figures 1, 2). Specifically, we used lentivirus-GCaMP3 and AAV1-GCaMP3 driven by the human synapsin promoter to image distinct cell types in the superficial OB. Although both vectors included the same promoter, each of them showed a very different expression profile. AAV1 densely infected cells with small soma and thin dendrites throughout the GL and granule cell layer, implying expression in inetrneurons (INs). In contrast, LV infected cells with large soma and thick dendrites mainly in the deeper parts of the GL, EPL and mitral cell layer, suggesting expression mainly in projection neurons. Examples for these distinct expression patterns in the GL are shown in Figures 1A,B (fixed tissue) and Figure 3A (*in vivo*).

To verify that these qualitative differences represent a real quantitative difference in the labeled cell types, we first carried out an extensive immunohistochemical characterization with a marker of OB excitatory neurons (Tbr2) as well as markers for different GL inhibitory INs (TH, CR and CB). Indeed, the vast majority of LV infected neurons expressed Tbr2 and rarely expressed any of the IN markers (Figure 1A). In contrast, the AAV1 cells were heterogeneous. ~45% of AAV1 infected cells expressed TH, a marker of GABAergic-Dopaminergic SA cells (Kiyokage et al., 2010), and the rest expressed Calretinin (CR), Tbr2, or were not labeled by any of the tested markers (Figure 1B).

To further validate the specificity of our data we restricted the imaging depth, such that LV infected cells were imaged at a depth of 130 ± 26 µm, while AAV1 infected cells were imaged at 82 ± 21 µm (Figure 2D). In addition, LV cells had significantly larger soma size compared with the AAV1 infected cells (Figure 2C). Taken together, the combination of excitatory neuron markers, large soma and deeper location suggested that the LV dataset contained mainly external and superficial TCs (which we collectively refer to as TCs) while the AAV1 sample is composed of mainly SA cells and different types of PGNs which we collectively refer to as GL-INs.

As ~25% of our GL-INs dataset included Tbr2+ excitatory neurons, we tested whether they have similar properties to the LV cells. To this end, we analyzed the calcium transient kinetics of both populations. The LV cells had homogenous transients with a fast decay constant (Figure 2E). In contrast, the AAV1 cells showed highly heterogeneous kinetics (Figure 2E), as expected from a mixture of different cell types. In addition, even the 25% of AAV1 cells with faster decay constant significantly differed from LV cells in their temporal dynamics during long odor stimuli (data not shown), a physiological property that clearly differentiated these datasets which is described in detail below. Thus, the distinct transient kinetics and the more superficial location suggest that the excitatory AAV1 cells are not TCs and may represent a subset of GL excitatory neurons (see also Aungst et al., 2003; Wachowiak and Shipley, 2006; Winpenny et al., 2011).

To label MCs, we used AAV5 expressing GCaMP5 under the CaMKII promoter, which labeled almost exclusively Tbr2 positive neurons with large soma in the mitral cell layer (Figures 1C, 2C,D). In a few experiments we also used transgenic mice expressing GCaMP3 in MCs (Chen et al., 2012). Mitral cells from both expression systems showed similar response profiles (Figure 3E, inset) and were pooled together.

### Basic odor response profiles of TCs, GL-INs and MCs

Following virus injection, mice were implanted with a chronic cranial window enabling access to large imaging regions with high optical quality and good mechanical stability (Adam and Mizrahi, 2011; Figure 2A). As a result, we were able to deliver relatively long odor protocols overcoming a common limitation in olfactory experiments. Our main protocol included 35 stimuli composed of seven monomolecular odorants, delivered for 1 s at four different concentrations (5–50 ppm, four trials), and for 15 s (50 ppm, four trials). In total, each field of view that we imaged underwent ~75 min of net imaging. To avoid contamination we used an olfactometer in which all odor channels were completely separated (Figure 2A). We used anesthetized animals in which numerous single neurons from single OBs could be studied longitudinally (i.e., many neurons with long odor protocols), while pinpointing their exact spatial location in 3D (Figures 2B,D). Altogether, single mice underwent up to 40 h of imaging (cumulative over several sessions), while mapping a substantial proportion of the dorsal surface of the OB at single cell resolution (Figures 2A,B). For clarity and since all three populations underwent the exact same protocol, we present the data herein for all three populations in parallel (we use the following color code: TCs-red, MCs-green, GL-INs-blue).

Under basal conditions, GCaMP fluorescence values (Δ*F/F*) were normally stable, while odor stimuli evoked transients readily apparent in all three populations (Figures 3A–C). In total, we collected and analyzed three full datasets: (1) 521 TCs (*n* = 6 OBs from five mice); (2) 397 MCs (*n* = 10 OBs from 10 mice); and (3) 1117 GL-INs (*n* = 6 OBs from four mice), at average depths of 130 ± 26 µm, 270 ± 41 µm and 82 ± 21 µm (mean ± SEM), respectively (Figure 2D). Tufted cells were more responsive (55%) as compared to the MCs (28%) and GL-INs (16%). Additionally, TCs responded consistently at lower concentrations (Figures 3B–D *T*-test, *P* < 0.001). Mitral cells showed relatively sparse and selective receptive fields with less than 12% of the cells responding to more than two odors in our panel (Figure 3E), consistent with previous studies (Davison and Katz, 2007; Fantana et al., 2008; Tan et al., 2010; Kikuta et al., 2013). As compared to MCs, TCs and GL-INs were more broadly tuned and significantly less selective with 32% of responsive TCs and 35% of responsive GL-INs responding to at least three odors (Figure 3E). Taken together, we identified two main differences among the three subpopulations: (1) TCs and GL-INs have wider response profiles as compared with MCs (Figure 3E); and (2) TCs respond at lower odor concentrations while MCs and GL-INs have a higher activation threshold (Figure 3D). While the general concept that MCs have narrow receptive fields and sparse responses has recently been shown (Tan et al., 2010; Kikuta et al., 2013), we now show that TCs are very different, showing low selectivity and low activation threshold.

### Functional architecture of TCs, MCs and GL-INs

Odors activate several glomeruli, forming discrete odor maps. Are odor maps retained downstream? If so, to what extent? If not, does any structure remain? To gain insight into these questions, we mapped the functional architecture of MCs, TCs and GL-INs. We imaged odor response profiles of the three populations to 28 stimuli (7 odors × 4 concentrations; Figure 3C). We imaged dozens of cells in single OBs (up to 317 per OB, chosen solely based on the optical clarity of the region), allowing us to cover inter-neuronal distances from a few microns up to 1.2 mm (Figures 2B,D, 4A–C, left panels).

To evaluate the functional organization of each population, we calculated pairwise signal correlations as a function of distance between all odor responsive neurons in each OB. Pairwise signal correlation (ranging from −1 to +1) measures the similarity between the average response profiles of two neurons and has been used in other sensory systems to describe structure-function relationships (Rothschild et al., 2010; Ko et al., 2011). Neurons were imaged in clusters such that correlations between nearby and distant pairs could be compared (Figures 4A–C). For example, three different OBs representing one example from each population are shown enlarged in Figure 4 (showing all 10 possible correlation values between all possible pairs). Given that TCs, MCs and GL-INs all receive their main input in glomeruli, we expected that nearby neurons would show high correlations and that correlations would rapidly drop with distance. Surprisingly, however, distinct cell types showed markedly different functional organizations (Figures 4D–F).

Tufted cells were, on average, highly correlated (0.63 ± 0.33, *n* = 8755 pairs; Figure 4D). Interestingly, high correlations were evident across all the spatial scales that we measured. In fact, correlation values between next door neighbors were often as high as of pairs 0.75 mm away (Figures 4A,D). Mitral cells were markedly different. Mitral cell pairs at all distances, including next door neighbors, were characterized by diverse correlation values. While the mean correlations moderately decreased with distance, the most obvious effect was that correlations of nearby MCs were highly variable. Highly correlated MCs within 100 µm (Figure 4B, bottom pair) were as prevalent as moderately correlated and uncorrelated MCs (Figure 4B, top left pair). The variable correlation structure of nearby MCs is consistent with previous imaging and electrophysiology studies. Specifically, MCs from different glomerular modules have been shown to intermingle in space and MCs from the same module (i.e., connected to the same glomerulus) could have distinct response profiles (Dhawale et al., 2010; Kikuta et al., 2013). Glomerular interneurons showed yet a different functional organization as compared to TCs and MCs. Correlations between GL-INs were high at short distances (within 100 µm) and sharply dropped with increasing distance (Figure 4F). The average pairwise correlation dropped from 0.76 ± 0.22 for nearby pairs (0–50 µm) to nearly zero for pairs that were >0.3 mm away (mean = 0.1 ± 0.22, *p* < 0.001, *T*-test). Thus, the correlation structure of GL-INs is consistent with a punctate activation pattern, as expected from sparse glomerular activation. While functional architecture of GL-INs and MCs can be intuitively explained based on previous findings, the TCs architecture was surprising. What then might underlie the unique functional architectures of TCs?

### TCs reliably encode ORN input

The high correlation values of TCs at long distances (Figure 4D) in conjunction with their higher sensitivity (Figure 3D) could mean that they are simply more easily recruited by odors due to lower activation threshold. Alternatively, the high correlation between distant TCs could indicate that they respond in a more global manner, such that distant glomeruli will contribute to their dense activation, possibly via lateral connections. To distinguish between these possibilities, we first carried out an experiment to test the latter possibility. We tested whether neurons responded only to local glomerular inputs by comparing their responses to different odors activating glomeruli on different parts of the dorsal surface of the OB, as measured by intrinsic signal imaging (e.g., Soucy et al., 2009). Thus, in addition to our main panel of seven odors (Figure 5A—“local” odors), we chose five additional odors that activated the OB mainly outside the imaging window, serving as an “external” odor set (Figure 5B). Three of these odorants (Ethyl lactate, Methyl Benzoate and Acetophenone) activated the lateral parts of the dorsal OB at the outskirts of the imaging window, whereas responses within the imaging window were minimal or absent altogether (Figure 5B). Two odorants (Butyl formate and r-Limonene) did not activate the dorsal surface at all (Figure 5B). Using this expanded odor set of 12 odors we collected calcium responses from 97 TCs, 94 MCs and 86 GL-INs (*n* = 5 OBs in total). All three populations responded to the “local odors” panel at the expected selectivity pattern (Figures 5C,D, compare to Figure 3E). Interestingly, none of the TCs, MCs or GL-INs responded significantly to any stimulus from the “external odors” set (Figures 5C,D). These data suggest that all three populations are driven largely by relatively local glomerular inputs (i.e., on the order of several hundreds of microns). Furthermore, these data show that the high correlation between TCs cannot be explained by promiscuous responsiveness to distant odors via long-range lateral connectivity.

Since lateral activation does not explain the TCs functional architecture, we next studied in detail the response profiles of the three neuronal populations with respect to the ORN inputs to the OB. To test this at the population level, we analyzed whether odors activating the same set of glomeruli would also tend to activate the same set of neurons. To obtain a large database of glomerular inputs, we collected intrinsic signal maps of the seven local odors from a different set of animals (*n* = 36 OBs). These maps were then processed to represent mainly the ORN component of the intrinsic signal (e.g., Meister and Bonhoeffer, 2001; Soucy et al., 2009, see Section Methods). The panel of seven local odors included odors with similar glomerular activation patterns (e.g., Methyl-Proprionate and Propanal) as well as dissimilar odors (e.g., Propanal and Ethyl Tiglate, Figures 5A, 6A). To quantify the similarity between the intrinsic signal maps, we calculated the Pearson correlation for all pairs of odor activation maps in each mouse (Figures 6A,D). As expected, the ORN input patterns of some odor pairs were similar, while others were not (Figures 6A,D). Correlation based cluster analysis revealed that the odorants were divided into three main clusters (Figure 6D, dendrogram).

To study the similarity in the population activity between responses to different odors, we plotted the population responses of each cell type for each odor (Figure 6B, for clarity only three cell-type odor ensembles are shown). We then calculated the ensemble correlation between all possible odor pairs (*n* = 21 pairs for each cell-type). Some odors evoked similar activation patterns by given sets of cells while other odorants activated completely different combination of cells. For example, almost every TC responding to M-Prop also responded to Prop (Figure 6B) resulting in very high ensemble correlation for this odor pair (*r* = 0.97, Figure 6C). In contrast, TCs responding to M-prop normally did not respond to E-tig (Figure 6B), thus this ensemble-pair was uncorrelated (*r* = 0.0, Figure 6C). The MC population responses were significantly more heterogeneous as compared to the TCs. For example, the MC population responses to Prop and M-Prop were 23% less correlated compared to TCs (*r* = 0.74, Figures 6B,C,E). Interestingly, the correlation between the dissimilar odors like M-Prop and E-Tig was markedly higher than in TCs (*r* = 0.34, Figures 6B,C,E). Population responses and correlation structure of GL-INs showed a similar pattern to those of TCs (Figures 6B,C,E). Ensemble correlation analysis for all odor pairs in the three neuronal populations is plotted as correlation matrices in Figure 6E. Notably, independent hierarchical clustering showed that ensemble correlations of TCs and GL-INs were divided to the same three clusters as those of the ORN signals (i.e., from the intrinsic signal imaging analysis). In contrast, in the MC population the three clusters were lost (Figures 6D,E, compare the dendrograms).

Having established the structure of correlation among ORN inputs (Figure 6D) and among the outputs of TCs, MCs and GL-INs (Figure 6E), we next compared between the ORN (inputs) and all three populations (outputs). Specifically, we plotted the similarity (i.e., correlation) of each odor pair at the input level as a function of the same value at the output level. The correlation between TCs outputs to glomerular inputs was high (*R* = 0.83, Figure 6F) and robust (see Section Methods), suggesting that if two odors activated the same glomeruli they will also activate the same ensemble of TCs. These data show that TCs faithfully reflect ORN inputs. Glomerular interneurons behaved similar to TCs although they had consistently weaker correlation values (Figures 6F,G). Notably, MCs did not show this strong input-output correlation, not even at the highest odor concentration (*R* = 0.46 at 50 ppm, Figure 6F). At intermediate and low odor concentrations (<25 ppm) MC ensemble activity was uncorrelated with ORN input (Figure 6G). This result supports the concept whereby MCs decorrelate similar odor inputs, and emphasizes the transformations odor representations undergo in the OB; as evident by the MC population activity.

**Figure 7:**
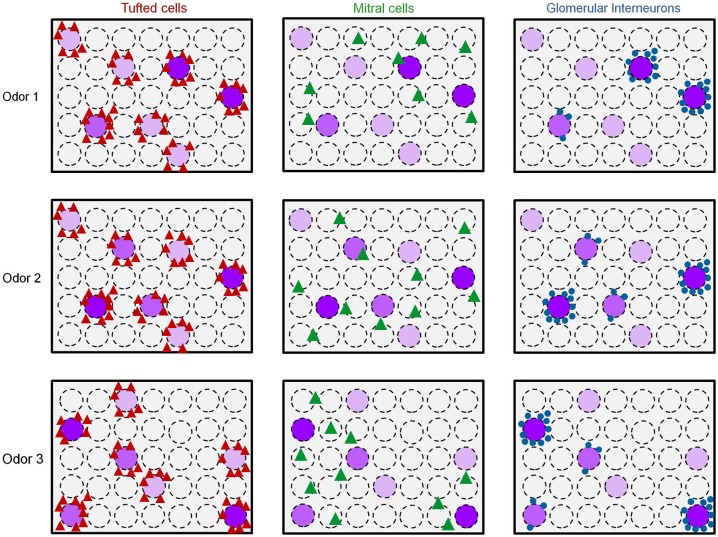
**Early spatiotemporal transformations in the olfactory bulb**. Scheme of the putative functional organization of the different cell types in the OB to distinct odors. Dashed circles represent glomeruli. Purple glomeruli are responsive to an odor, and the purple color intensity represents the strength of glomerular activation. Odor 1 and odor 2 induce similar glomerular activation patterns while odor 3 activates a different set of glomeruli. Red triangles = Responsive TCs, Green triangles = Responsive MCs, Blue circles = responsive GL-INs.

Taken together, these results offer an explanation to the distinct functional architecture of GL-INs and TCs shown in Figure 4. Namely, GL-INs are active around responsive glomeruli but biased to a small subset of those, possibly the strongly activated ones (Figure 7). Tufted cells, on the other hand, are more sensitive and respond to ORN activity, with little transformation. Notably, the TCs output is a near-perfect reflection of glomerular activity, be it strongly or weakly activated glomeruli (Figure 7). The high correlations of distant TCs are thus explained by the fact that several glomeruli within our imaging window respond to the same odors (see e.g., Figure 5A). Mitral cells, in contrast, present a sparse and highly transformed activation map. In MCs, distinct spatial activation patterns are evident even for similar odors (e.g., Odor 1 vs. Odor 2 in Figure 7). Figure 7 provides a schematic summary of our results thus far showing spatial activation profiles of the three populations in response to three putative odor maps. We next turned to analyze how these distinct populations behave in the temporal domain.

### Persistent odor stimuli evoke distinct temporal dynamics in TCs and similar dynamics in GL-INs and MCs

Given that mice can discriminate between odors with single sniffs (Uchida and Mainen, 2003; Abraham et al., 2004; Smear et al., 2011), 1 s stimuli already contain plenty of information for perceptual decisions and computations therein (Bathellier et al., 2008; Shusterman et al., 2011; Smear et al., 2011). However, in the real world, sensation and perception are affected by longer time scales too, reflecting the continuous or slowly changing chemical environment. Studying long stimuli is important for understanding computations such as adaptation and figure-background separation (Dalton, 2000). But, how the olfactory system deals with long odor flow is still not well understood. Thus, we next characterized the response profiles of TCs, MCs, and GL-INs during persistent odor exposure.

**Figure 8:**
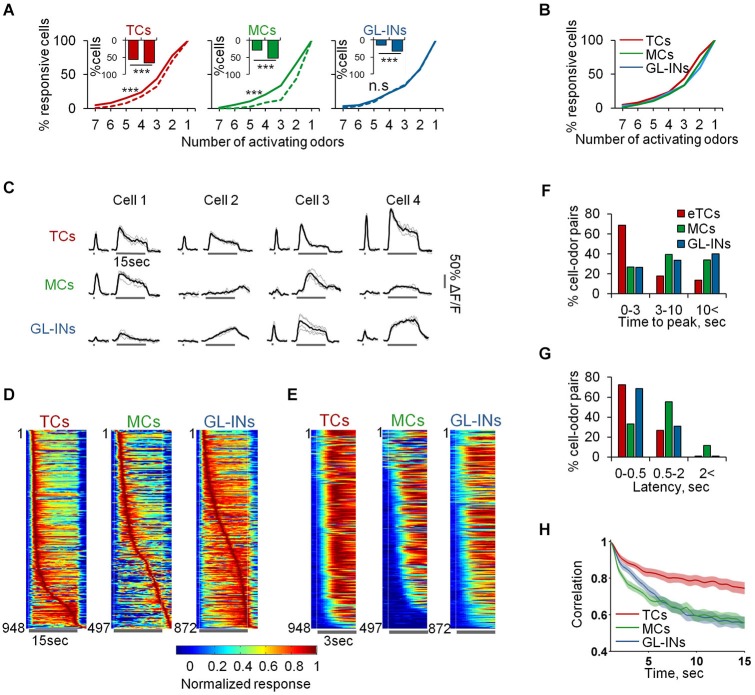
**Slow temporal dynamics of TCs, GL-INs and MCs. (A)** Odor selectivity graphs for 1 s (dashed) vs. 15 s (solid) stimuli (***p < 0.001, Kolmogorov-Smirnov test). Inset shows the responsiveness at 1 (left bar) vs. 15 (rightbar) second odor stimulus. All populations are more responsive for the longer stimulus (***p < 0.001, Chi^2^ test). **(B)** Odor selectivity graph for 15 s stimuli only. Same as solid line in **(A)**. **(C)** Examples for traces of cell odor pairs at 1 and 15 s odor stimuli. All traces show the responses of different cells from a single OB to the same odor (Butanal). Gray—single trials, Black—mean response. **(D)** Time course for all the responsive cell-odor pairs, normalized to the peak and sorted by the time to peak. **(E)** Time course for all the responsive cell-odor pairs, normalized and sorted by the response latency. **(F)** Distribution of the time to peak, corresponding to the data in **(D)**. **(G)** Distribution of the response latency, corresponding to the data in **(E)**. **(H)** Correlation of the cell ensembles with the initial response pattern over time, averaged across all odors and OBs (mean ± SEM).

Response profiles to persistent odor stimuli (15 s long) revealed distinct spatio-temporal response profiles between the three subpopulations. First, we compared the spatial extent of activation following 15 s long stimuli as compared to 1 s stimuli. As expected, more neurons were recruited when longer stimuli were used. There was a larger increase in the responsiveness of the population of GL-INs and MCs but only a modest increase in TCs activation (110% and 85% increase vs. 20% increase, Figure 8A, insets). Interestingly, despite the increased responsiveness of GL-INs, their average response selectivity remained the same (Figure 8A—GL-INs). The selectivity of TCs decreased only slightly (Figure 8A—TCs) while MC responses became significantly less selective upon long odor exposure (Figure 8A—MCs). Consequently, under persistent odor stimuli all three populations became equally selective (Figure 8B). These results suggest that a larger portion of the OB network is recruited to participate in odor coding during persistent stimuli.

Next, we compared response dynamics of the same cell-odor pairs to 1 s and 15 s odor stimuli (Figure 8C). Different TCs were remarkably similar in their response dynamics. Namely, TCs showed a fast phasic response followed by decay to a weaker tonic state. This response pattern can be seen in the individual calcium responses, and across the population (Figures 8C–E). Over 70% of TCs cell-odor pair responses peaked within 3 s and adapted quickly (Figures 8D–F—TCs). We did not detect any “off” responses in almost 1000 eTC-odor pairs. Mitral cells and GL-INs, in contrast, showed distinct and highly heterogeneous temporal dynamics. Some responses were adaptive, while others were stable or showed slow rise and slow decay (Figures 8C–F). Some cells were even characterized by a persistent increase in their responses during the long stimulus (Figures 8C,D). Quantitatively, only ~25% of the responses of these populations reached their peak within 3 s, while most response maxima were further delayed (Figure 8F). As expected, MCs occasionally showed off-responses (Davison and Katz, 2007; Kato et al., 2012), but such responses were rarely observed in GL-INs (Figure 8D). The traces in Figure 8C were all taken from single OBs and show responses to the same odor, demonstrating that both MCs and GL-INs from the same OB display rich temporal patterns. At the population level, the rich temporal dynamics resulted in decreased correlation of the MCs and GL-INs ensembles with their initial response pattern over time (Figure 8H). This observation is consistent with previous work showing MC decorrelation in mice (Kato et al., 2012) and fish (Friedrich and Laurent, 2001), and now extended to GL-INs as well. As expected from the TCs’ homogenous response patterns (Figures 8C,D), TCs ensembles remained relatively correlated throughout the stimulus (Figure 8H). Interestingly, GL-INs and TCs shared similar latencies of their calcium transients, which were fast, with latencies of normally less than 0.5 s (Figures 8E,G). Mitral cells transients started later, usually between 0.5 to 2 s after the odor onset, or even later during the stimulus (Figures 8E–G). Taken together, these data suggest that TCs form not only a reliable reflection of the ORNs spatial organization, but also reliably transmit information about odor onset. Upon long odor stimulation new MCs with rich temporal patterns are recruited to the population response. The behavior of GL-INs over time (i.e., their partial similarity to MCs responses during long stimuli) suggests that they modulate MCs but not TCs.

### Spatiotemporal response patterns

**Figure 9:**
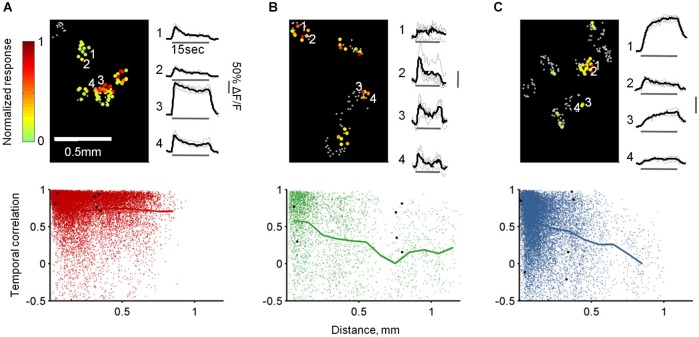
**Spatiotemporal response patterns of TCs, GL-INs and MCs. (A)**—TCs, **(B)**—MCs and **(C)**—GL-INs. Top left—activation map from a single OB for a single odor (Butanal). Each cell is denoted by a dot, gray dots represent non-responsive cells, color-code represent the peak ΔF/F for 15 s stimulation with Butanal. Responses in each bulb were normalized to the highest response. Top right—example of the response traces for four cells (marked by a number on the map). Gray—single trials; black—mean response. Bottom—correlation of the response patterns of all pairs of cells responding to the same odor in each bulb (up to seven odors for a pair), plotted as a function of the distance between the cells (TCs, *n* = 19918 pairs from six OBs, GL-INs—16383 pairs from six OBs, MCs—3229 pairs from 10 OBs). Thick line—mean correlation binned every 100 µm. Black dots—correlation between all possible pairs of the cells from the examples in the top panels.

Finally, we studied the combined spatial and temporal patterns of each population by calculating the similarities between the temporal response patterns of all cells responding to the same odor in each OB (Figure 9, top panels). Specifically, we calculated all pairwise correlations between the traces, and then plotted these values as a function of the distance between the cells. As expected, TCs at all distances responded similarly to the same odor reflecting their faithfulness to the stimulus in space and time (Figure 9A). In contrast, MC responses were heterogeneous at all distances, although along long odor presentation nearby cells had higher probability to respond similarly (average *R* = 0.62 ± 0.35 at 0–50 µm distance compared with *R* = 0.34 ± 0.44 at 500–550 µm distance, *p* < 0.0001, *T*-test, Figure 9B). Notably, GL-INs’ temporal patterns were heterogeneous at all distances even for nearby pairs. These heterogeneous patterns were also evident when calculated only between pairs of cells imaged simultaneously in the same imaging plane (data not shown) suggesting that nearby GL-INs (with high probability to be connected to the same glomerulus) can process the same odor with distinct temporal patterns.

## Discussion

In this study, we revealed that the ORN input map in the OB is transformed into separate sensory maps. One map, that of the TCs, resembles a labeled-line representation of glomerular activity in space and time. A second map of MC outputs is a transformed version of the ORN map in both space and time. We also imaged GL-INs activity which similar to TCs reflects the ORNs map. However, GL-INs representations are less sensitive and more punctuate compared with TCs.

### Two parallel output streams from the OB to the cortex

Mitral cells and TCs have been studied by single neuron anatomy and physiology. Single neuron tracing showed that MCs and TCs project into separable cortical regions (Nagayama et al., 2010; Igarashi et al., 2012), and single neuron recordings show that they differ in their fast temporal response properties to odor stimulation (Fukunaga et al., 2012; Igarashi et al., 2012). Both of these properties suggest that MCs and TCs convey distinct information streams to the cortex. Our data support and extend the observations of single neuron studies by showing two separate streams of information at the population level. Importantly, we describe how topographic information is conveyed by each of these parallel output streams.

The TCs stream conveys a relatively undistorted version of glomerular identity including its precise time of activation. Based on the known connectivity patterns of TCs (Hayar et al., 2004; Wachowiak and Shipley, 2006; Gire et al., 2012; Igarashi et al., 2012) this information could reach locally (to all GL-INs as well as MCs), as well as to more specific regions in the anterior olfactory cortex. Thus, both local neurons as well as neurons of the anterior olfactory cortex may make use of the precise information of the topographic map. The simplest interpretation of our data is that TCs function as precise broadcasters of ORN activity. Our data imply that TCs may function as a simple relay station. At the level of the local OB network this may seem redundant because the same information is already conveyed by the ORNs themselves. However, relaying reliable signal for further use could be an essential property for neural circuits. Convergence of thousands of ORNs onto dozens of synchronized TCs (Hayar et al., 2005; Wachowiak and Shipley, 2006) may reduce the noise and increase the fidelity of the sensory signal. Further, TCs show a toggle-like behavior with fast on/off kinetics (Figure 8), suggesting that they carry information about precise timing of odors in the environment. This is important information to have for any decoder downstream not only as sensors of changes in odor environments but also as a synchronization signal with other processing channels. The reliable odor identity conveyed by TCs also suggests that their dominant synaptic input comes from ORNs. Inputs from other neurons, like PGNs, could also act to inhibit TCs in a recurrent manner (Gire and Schoppa, 2009; Shao et al., 2009). These inhibitory connections may contribute to other aspects of the response profile of TCs like their rapid adaption seen in longer stimuli (Figure 8). Finally, TCs have low thresholds (Figure 4) and could serve as amplifiers of weak ORN inputs, thus contributing to gain control mechanisms (Cleland, 2010; Murthy, 2011).

In marked contrast to the TCs, the information conveyed by the MC population is complex in both space and time. The role of MCs in odor coding has been studied extensively by electrophysiology and imaging, showing their sparse response profiles and complex temporal patterns (Davison and Katz, 2007; Fantana et al., 2008; Cury and Uchida, 2010; Tan et al., 2010; Shusterman et al., 2011; Kikuta et al., 2013). In addition, MC activity has been shown to decorrelate similar odor inputs; a process thought to support odor discrimination behavior (Cleland, 2010; Murthy, 2011; Friedrich, 2013). In fish, early in the response, similar odors activate similar sets of MCs but these then rapidly decorrelate (Friedrich and Laurent, 2001; Yaksi et al., 2007; Friedrich, 2013). A similar process has been implied in mice (Kato et al., 2012). Our data also show deccorelation but through a different mechanism. Mitral cells distort the topography of the ORN map; a distortion that was evident in both low and high odor concentrations (Figure 6). This argues that decorrelation in space and time is a fundamental transformation carried out in the OB by MCs (Cleland, 2010; Friedrich, 2013), but not by TCs.

Taken together, our data show that two similar odors will activate similar ensembles of TCs but distinct MC patterns (Figures 6, 7-odors 1 and 2). Mitral cells are thus efficient for encoding fine changes in odor identity and are useful for accurate discriminations (Figure 6). Tufted cells may not be as efficient for fine discrimination, but their lower activation threshold (Figure 3, see also Igarashi et al., 2012) and shorter latency (Figure 8, see also Fukunaga et al., 2012; Igarashi et al., 2012), suggest that TCs inform the cortex about precise temporal aspects of the stimulus in a fast and sensitive manner.

### Division of labor in the GL

Unlike MCs, eTCs which are presumably a major part of our TCs dataset are also involved in local processing (Hayar et al., 2004; Wachowiak and Shipley, 2006; Gire et al., 2012; Igarashi et al., 2012). Extensive characterization by slice physiology showed that eTCs are mono-synaptically driven by ORNs and provide excitation to most cells within the GL (Gire et al., 2012). In addition, it was recently suggested that TCs (as opposed to ORNs) are the main source of excitation to MCs (Gire et al., 2012). Considering that MCs and TCs send their main dendritic tuft into the same glomerulus, a relay between TCs and MCs seems inefficient. In turn, this raises the possibility that other bulbar neurons receive the same information MCs do in an orchestrated manner. This further signifies the role of temporal precision in the OB that is mediated by TCs.

Similar to TCs, GL-INs have wider response profiles as compared with MCs (Tan et al., 2010; Kikuta et al., 2013; Figure 3). In addition, the response profiles of both TCs and GL-INs are correlated with ORN inputs (Figure 6), suggesting that both populations are driven by intraglomerular mechanisms. Nevertheless, TCs and GL-INs differ in several important ways, which impact their contribution to odor transformations. First, GL-INs are driven directly by ORNs, by eTCs, by MCs or by other GL-INs (Wachowiak and Shipley, 2006; Shao et al., 2009). Despite this wide platform of activation, GL-INs are much less responsive compared to TCs, and on average respond to higher odor concentrations, implying that GL-INs follow only the strongly activated glomeruli (Figures 3, 7). Second, when GL-INs are activated they seem to act unanimously as a glomerular unit (Figure 4E, see also Homma et al., 2013). The role of this unanimous activity at selected glomeruli is unclear. On one hand, this can “self-inhibit” glomerular information. On the other hand, it can inhibit (or excite) neighboring glomeruli. Either option depends on the wiring specificity of the neurons that belong to a single glomerulus, but this information is still unknown. Another level of complexity stems from the fact that GL-INs are molecularly and functionally diverse. Glomerular interneurons contain various types of inhibitory PGNs (Parrish-Aungst et al., 2007), dopaminergic (DA) SA cells (Kiyokage et al., 2010) and possibly some excitatory INs (Aungst et al., 2003; Wachowiak and Shipley, 2006; Winpenny et al., 2011), all of which are continuously replaced via adult neurogenesis and contribute differentially to odor coding (Lledo et al., 2006; Adam and Mizrahi, 2010, 2011; Livneh et al., 2014). Notably, our sample does not equally represent the full scope of heterogeneity of this population (Figure 1). In fact, a large fraction of our GL-INs dataset contains GABAergic-dopaminergic INs that were shown to form lateral connections within the GL (Kiyokage et al., 2010; Liu et al., 2013). Our data suggesting that GL-INs function at strongly activated glomeruli, may imply that those are DA neurons that mechanistically contribute to MCs decorrelation via inter-glomerular lateral inhibition.

### MCs provide additional information at slow temporal scale

How odor information processing changes during long odor exposure is a central, yet poorly studied question in olfaction (Dalton, 2000). We provide initial insights into this issue by showing that MC responses are temporally rich and undergo slow temporal modulations (Figures 8, 9). This result suggests that the MC population conveys to the cortex additional information as animals continuously sniff. Tufted cells however, are different. At persistent odor exposure, TCs show strong phasic activation followed by fast adaptation of the response (Figure 8). This adaptation suggests that TCs are prominent in conveying the onset of odor stimuli. Tufted cells adaptation may be a result of feedback inhibition from PGNs (Gire and Schoppa, 2009; Shao et al., 2009), or a simple reflection of adaptation at the ORN level (Zufall and Leinders-Zufall, 2000). The weak steady state response of TCs shows that spatial odor information continues to flow downstream steadily, but that precise temporal information is lost. Glomerular interneurons also go through slow temporal modulations of their responses. We speculate, then, that the slow temporal transformations of odor information at the MC level may be caused by GL-INs. This argument is further supported by the observation that nearby GL-INs often responded to the same odor with distinct temporal dynamics (Figure 9).

### Single cell calcium imaging in the OB

Population calcium imaging is a powerful tool to study neural circuits, particularly with advances in genetically encoded indicators. This method, however, still has considerable caveats, like poor temporal resolution and lower sensitivity as compared to electrophysiology. Nevertheless, calcium indicators are fair proxy for average spiking activity, they allow to record from many cells simultaneously, and they have the advantage of stable and long recording time. Here, we used these advantages to densely sample the tissue, covering four orders of magnitude in spatial resolution. Additionally, we could study slow physiological processes, which are more difficult to carry out with some electrophysiological methods given the lengthy odor protocols that are used. We utilized these advantages and imaged OBs in anesthetized mice. Dense mapping benefits tremendously from a stable preparation which can be achieved in anesthetized animals with relative ease (each of our mice spent dozens of hours under the microscope). Recent data suggests that anesthesia can change the activity in the OB to varying extents (Rinberg et al., 2006; Kato et al., 2012; Wachowiak et al., 2013). Although we cannot completely rule out the effects of anesthesia, recent data showed that the temporal differences between MCs and TCs seems to be preserved under anesthesia (Fukunaga et al., 2012). We expect that the advantages of imaging will continue to complement electrophysiology in deciphering the complex computations taking place already at the very initial stages of odor processing.

## Conflict of interest statement

The authors declare that the research was conducted in the absence of any commercial or financial relationships that could be construed as a potential conflict of interest.

## Footnotes

^1^http://imagej.nih.gov/ij/
